# Open-source lower controller for twelve degrees of freedom hydraulic quadruped robot with distributed control scheme

**DOI:** 10.1016/j.ohx.2022.e00393

**Published:** 2023-01-03

**Authors:** Lizhou Fang, Junhui Zhang, Huaizhi Zong, Ximeng Wang, Kun Zhang, Jun Shen, Zhenyu Lu

**Affiliations:** a Zhejiang University, Hangzhou, Zhejiang Province, China; b Nanchang University, Nanchang, Jiangxi Province, China

**Keywords:** Hydraulic quadruped robot, Digital signal processing, Controller, Automatic code generation

## Abstract

Nowadays, hydraulic quadruped robot shows high power density, good impact resistance and robustness in the research. The controller is the key to realize these features. This paper shows the design of an open-source single-leg controller for the hydraulic quadruped robot Spurlos using a distributed control scheme. The single-leg system of the hydraulic quadruped robot Spurlos contains three angle encoders, three servo valves and six pressure sensors, which has the same components as most single-leg systems. Through the chips designed in the controller, the signal can be received from the encoders and the sensors, meanwhile the signal can be delivered to the servo valves. The software part of the controller adopts the MBD (Model-Based Design) method, which can greatly improve the development efficiency. According to the experiments, the controller design is reasonable, stable operation, and can satisfy the requirements of the hydraulic quadruped robot for leg motion control. The controller designed in this paper provides a solution to the problem that there is no ready-made control board for hydraulic quadruped robot which have three degrees of freedom for each leg. It enables the control researches for hydraulic quadruped robots to be more easily implemented.

## Graphical abstract

**Figure:**
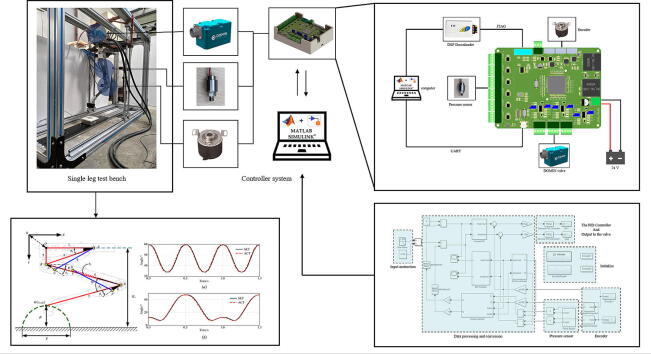

## Specification table:

**Table:** 

Hardware name	Quadruped robot controller
Subject area	Educational tools and open source alternatives to existing infrastructure
Hardware type	Mechanical engineering and materials science
Open Source License	CERN OHL
Cost of Hardware	103.70 USD
Source File Repository	https://doi.org/10.17632/zym6bwk37h.2

## Hardware in context

In recent year, the research on legged robots has concentrated on quadruped robots. Express delivery, manufacturing inspection, and load transfer are all major applications for quadruped robots. According on the kind of drive, quadruped robots may be classified as either electric or hydraulic, with hydraulic quadruped robots having the benefits of a high-power density, a large load capacity, good safety and dependability, and good impact resistance (see Table 1).

**Table 1:** The signals type required in hardware.

Signals	Voltage	Type	Number
Pressure sensor input signal	10 V	0–20 mV	6
Encoder input signal	24 V	CAN	3
Valve control output signal	24 V	4–20 mA	3
Upper computer input signal	Null	CAN	1
Serial port debugging	Null	UART	1

Pricing Notes: price will vary with source.

Some researchers have designed the controllers for quadruped robots; the primary control schemes for these controllers are centralized and distributed designs [1]. The BigDog hydraulic quadruped robot designed by Boston power company uses a centralized control scheme. It uses the PC104 industrial control computer running QNX (quick Unix) as the robot's control system to realize its motion control, motion planning, attitude estimation, and environmental awareness [2], [3]. The hydraulic quadruped robot HyQ designed by IIT (Italian Institute of Technology) uses the PC104 industrial control computer running the Linux (xenomai) real-time operating system as the robot's control system and is matched with five data acquisition boards to realize the control of the robot [4], [5], [6], [7], [8], [9]. This scheme uses a single core controller to control the whole quadruped robot, which has high real-time control. So, it requires this controller's high operational performance and other acquisition cards to complete the sensor data reading. The SCalf hydraulic quadruped robot designed by Shandong University uses a distributed control scheme, which separates the upper controller from the lower controller of the robot [10]. It uses different controllers to control the robot separately. The upper controller needs to perform such tasks as attitude control, gait planning, user command receiving and processing, and communication with the lower controller; the functions to be realized by the lower controller mainly include single leg sensor signal acquisition, single leg actuator control, and single leg control strategy implementation. This control scheme assigns different control tasks to different controllers more rationally, which makes the control framework of the hydraulic quadruped robot clear and reduces the cost of the controller. It will become the most mainstream control scheme in the future. Still, it also needs a more integrated modular design to optimize the speed and efficiency of data interaction between various parts (see Table 2).

**Table 2:** Design files summary.

Design file name	File type	Open source license	Location of the file
Quadruped robot controller.PRJPCB	PRJPCB	CERN OHL	https://data.mendeley.com/datasets/zym6bwk37h/2
Quadruped robot controller_SCH.SCHDOC	SCHDOC	CERN OHL	https://data.mendeley.com/datasets/zym6bwk37h/2
Quadruped robot controller_SCHLIB.SCHLIB	SCHLIB	CERN OHL	https://data.mendeley.com/datasets/zym6bwk37h/2
Quadruped robot controller_PCB. PcbDoc	PcbDoc	CERN OHL	https://data.mendeley.com/datasets/zym6bwk37h/2
Quadruped robot controller_PCBLIB.PcbLib	PcbLib	CERN OHL	https://data.mendeley.com/datasets/zym6bwk37h/2
Quadruped robot controller_PCB.STEP	CAD	CERN OHL	https://data.mendeley.com/datasets/zym6bwk37h/2
Protective shell (base).STEP	CAD	CERN OHL	https://data.mendeley.com/datasets/zym6bwk37h/2
Protective shell (cover).STEP	CAD	CERN OHL	https://data.mendeley.com/datasets/zym6bwk37h/2
BOM.xlsx	excel	CERN OHL	https://data.mendeley.com/datasets/zym6bwk37h/2
Test_track.slx	Simulink	CERN OHL	https://data.mendeley.com/datasets/zym6bwk37h/2

**Fig. 1:**
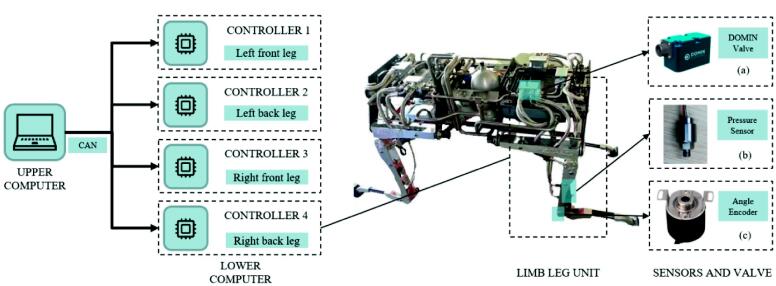
The control schemes and sensors on the limb leg unit of quadruped robot Spurlos: (a) DOMIN rotary direct drive servo valve. (b) Hydraulic cylinder pressure sensor. (c) Angle encoder.

The hydraulic quadruped robot Spurlos' control strategy is chosen as the distributed design scheme (Fig. 1). The upper computer controls the robot's motion planning, posture control, and gait control, and the lower computer controls every single leg. This work is the lower single-legged execution layer controller in the control scheme, so only the signal acquisition and control of the single leg of the quadruped robot need to be considered. The degrees of freedom of the Spurlos limb leg unit include hip abduction/adduction, hip flexion/extension, and knee flexion/extension. Each degree of freedom is controlled by a hydraulic cylinder that our research group designs. In the limb leg unit, each hinge is controlled by a rotary direct-drive servo valve (4–20 mA analog current, Fig. 1-a), each hydraulic cylinder has two hydraulic pressure sensors that detect the pressure level of the left and right chambers (analog signal, Fig. 1-b) and each hinge carries an angle encoder (Control Area Network communication, CAN communication, Fig. 1-c). This controller was designed according to the above requirements, including several modules such as the sensor data acquisition module, servo valve drive module, and communication module. Model-based design (MBD) method is adopted in the software part, which can significantly improve the development efficiency. The controller circuit board's protective housing and heat dissipation scheme were designed according to the circuit board shape. This controller can fit with any hydraulic quadruped robot that meets the above composition (see Table 3).

**Table 3:** The bill of materials about the protective shell.

Designator	Component	Number	Cost per unit -currency	Total cost -currency	Source of materials	Material type
Screws	M3*14 Stainless Steel Socket Head Screws	8	0.03048	0.2438	SZLCSC	Stainless Steel
Copper stud	M3*15 Double female head copper stud	4	0.2438	0.9754	SZLCSC	Brass
Cooler	35*10*35 mm Aluminum profile radiator with fixing lug	1	0.3048	0.3048	SZLCSC	Aluminium Alloy
Cooling fan	40*40*10 mm Delta Cooling Fan 24 V	2	1.8288	3.6576	SZLCSC	

## Hardware description

Our board offers the following features:

• The function of controlling the limb leg unit of the Spurlos hydraulic quadruped robot is complete, and other hydraulic quadruped robot that meets the same composition.
• The output impedance can be adjusted by adjusting the potentiometer
• MCU (Microcontroller Unit) clock frequency up to 150 MHz
• The DAC (Digital to Analog Converter) chip has 4-channel 16-bit conversion accuracy
• The ADC (Analog to Digital Converter) chip has 8-channel 16-bit conversion accuracy and the sampling rate is up to 115 khz
• The software is designed as Simulink block diagram modeling in MATLAB, which is simple and easy to use, and has external simulation mode

**Fig. 2:**
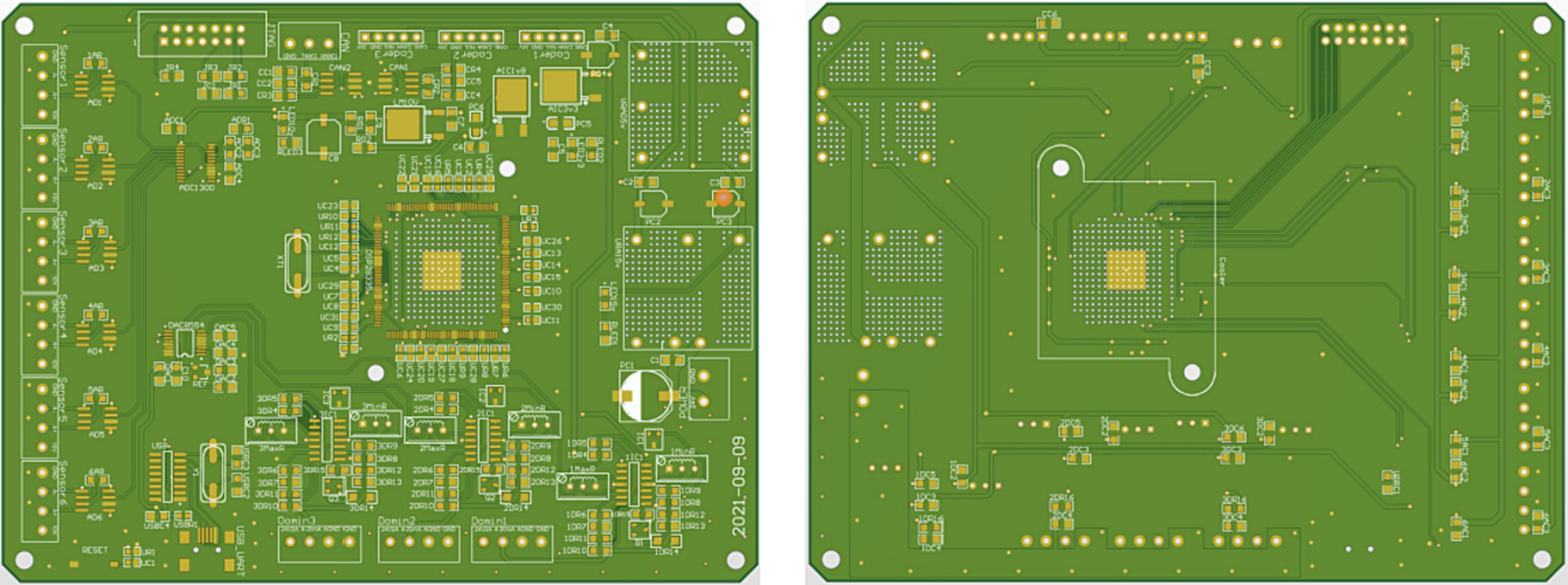
The Front and reverse side of the PCB.

To complete the above features, the hardware design needs to be able to send the following types of signals:

The printed circuit board (PCB, Fig. 2) was designed using Altium Designer. Considering that a large amount of data operation is required for the robot’s single-leg controller, and the single-leg control frequency is at least 1000 Hz to maintain the stability of leg operation, the controller finally selects TI's (Texas Instruments) high-performance TMS320F28335 as the 32-bit core processor, with a clock frequency of up to 150 MHz, 256 K 16-bit FLASH and 34 K 16-bit SRAM [11]. The TMS320F28335 generates a lot of heat when it is in use, so it needs to consider adding a heat sink when designing the housing and an additional heat sink when designing the housing. A fan carries out the external heat dissipation.

**Fig. 3:**
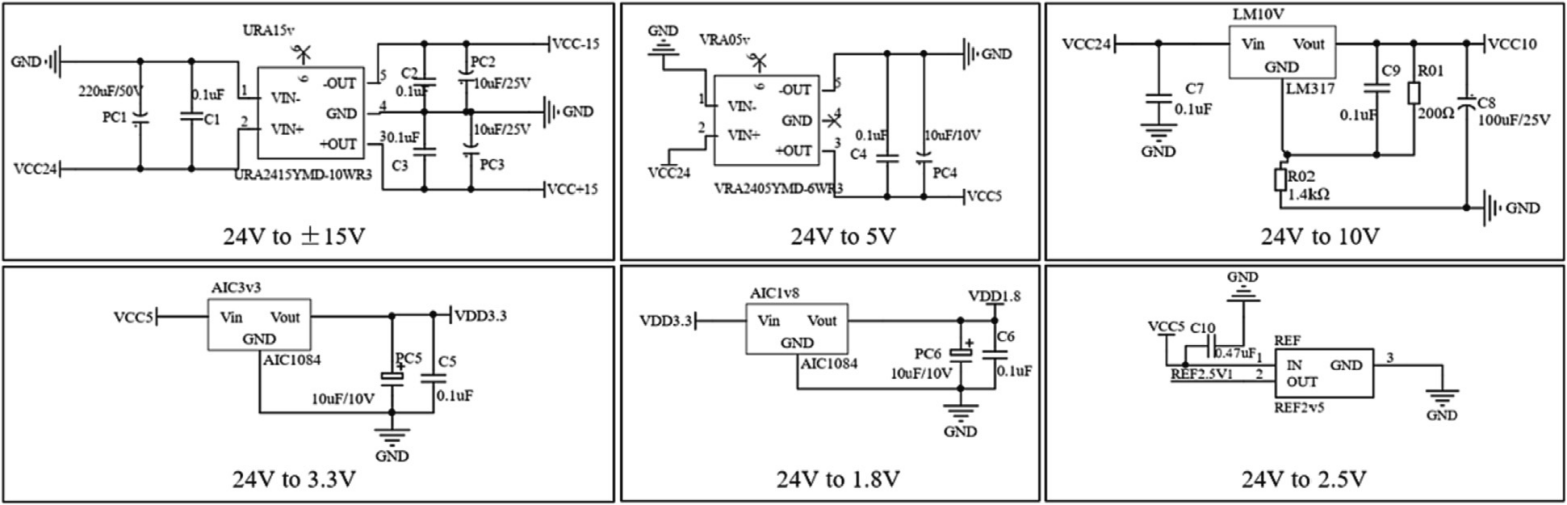
The power supply module schematic.

This circuit board needs a stable 24 V power supply for power supply. To power the DSP (Digital Signal Processing) master and the ADC and DAC chips, the supply voltage needs to be converted to 5 V, 3.3 V, and 1.8 V output; for an operational amplifier, the supply voltage needs to be converted to ±15 V output; for the fluid pressure sensor, the input voltage needs to be converted to 10 V output. At the same time, to ensure the stable output of the DAC8554, a 2.5 V reference voltage is also required. In summary, the board's power supply module needs to convert the 24 V input voltage to ±15 V, 10 V, 5 V, 3.3 V, 2.5 V, and 1.8 V outputs, so it needs the corresponding six power conversion circuits are shown below (Fig. 3), power supply module schematic). Because the number of voltages used by this board is large, the voltage conversion modules are selected to meet the performance of the case as far as possible to choose a simpler peripheral circuit chip to reduce the board size in the power supply part of the ratio and complexity.

**Fig. 4:**
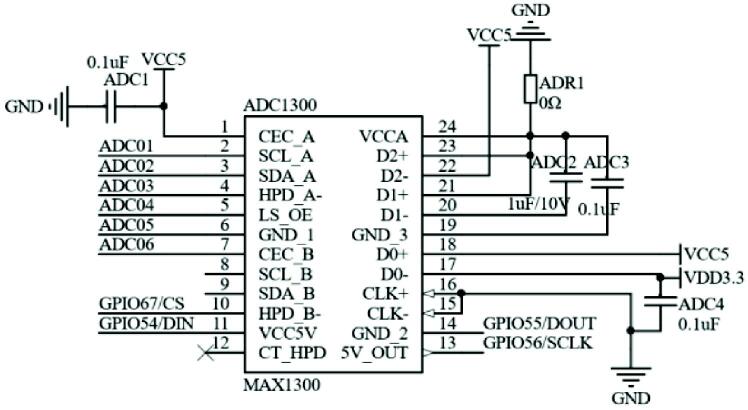
The analog-to-digital conversion circuit schematic.

Since the precision of TMS320F28335′s built-in A / D conversion unit is only 12 bits, the accuracy is low. The input range is small, which can’t satisfy the input requirements of fluid pressure sensor detection. The controller selects MAX1300 as the external A / D conversion chip, which has 8-channel 16-bit conversion precision and sampling rate up to 115khz, which meets the design requirements, communicates with DSP through SPI (Serial Peripheral Interface) bus, and selects the conversion channel through chip selection pin and control command. The analog-to-digital conversion circuit is shown in Fig. 4 [12], [13], [14].

**Fig. 5:**
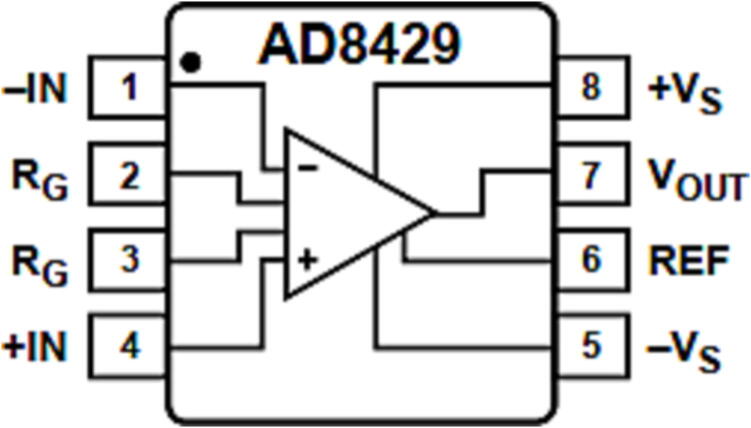
The chip pin distribution of AD8429.

Due to the small input signal range of the pressure sensor, it is necessary to amplify the signal—select the AD8429 instrument amplifier for amplification. Compared with a general operational amplifier, it has the advantages of a high standard mode rejection ratio, small linear error, and low noise. The chip pin distribution is shown in Fig. 5 [15].

The transfer function of AD8429 is:

(1) $$V_{\mathrm{out}}=G\times \left(V_{\mathrm{in}+}+V_{\mathrm{in}-}\right)$$

In which the magnification is:

(2) $$G=1+\frac{6k\Omega }{R_{G}}$$

LetG=500, to get$R=\frac{6k\Omega }{G-1}=\frac{6k\Omega }{500-1}=12.1\Omega \approx 12\Omega$.

**Fig. 6:**
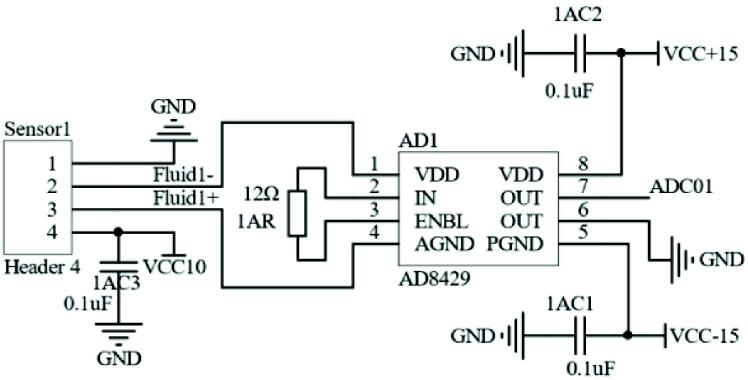
The AD8429 circuit schematic.

Therefore, the circuit of AD8429 is shown in the Fig. 6. It meets the expected amplification effect.

**Fig. 7:**
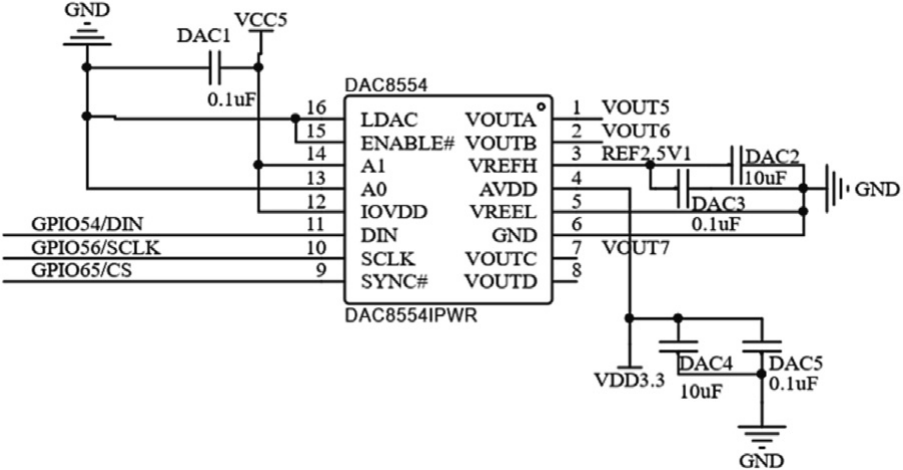
The digital-to-analog conversion circuit schematic.

To output, the 4–20 mA analog current required by the servo valve, a D / A conversion circuit design is required. The DAC8554 is selected for digital-to-analog conversion, which communicates with the DSP through the SPI bus and has 4-channel 16-bit conversion accuracy to meet the requirements. Its digital-to-analog conversion circuit is shown in Fig. 7 [16].

**Fig. 8:**
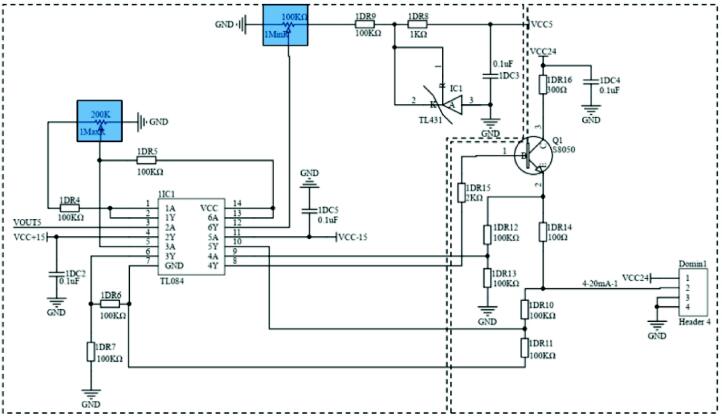
The voltage to current conversion schematic. Left: The output voltage amplification circuit of operational amplifier TL084 adjusts the output impedance through MINR and MAXR (blue). Right: The triode current amplifier circuit. (For interpretation of the references to colour in this figure legend, the reader is referred to the web version of this article.)

Due to the output current of DAC8554 being small and not enough to drive the servo valve, voltage to current conversion is required. Firstly, the output voltage is amplified by operational amplifier TL084, and then the current is amplified by triode, as shown in the following circuit; among them, the sliding rheostat MINR and MAXR can be adjusted to match the output impedance (Fig. 8).

**Fig. 9:**
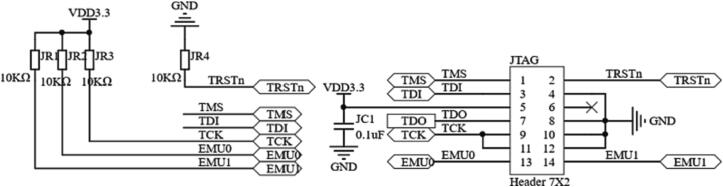
The JTAG circuit schematic.

The JTAG (Joint Test Action Group) interface is reserved on the controller for program download and debugging, and its circuit is shown in Fig. 9.

**Fig. 10:**
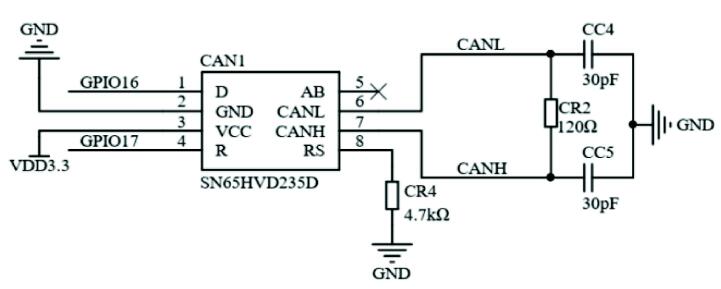
The CAN communication module schematic.

The controller needs to design a communication module to receive instructions from the upper computer. Since the quadruped robot has four legs, selecting an industrial bus scheme that can carry out multi-node communication is necessary. The commonly used bus communication schemes include CAN bus, RS485 standard bus, and industrial Ethernet (EtherCAT). Considering the model of the core processing chip, the TMS320F28335 has CAN communication pin and UART (Universal Asynchronous Receiver/Transmitter) serial communication pin, which can carry out CAN bus communication and RS485 standard bus communication, respectively, without customizing the communication sequence. Considering that the serial port needs to be connected with the user's computer to collect data later if the upper computer communicates simultaneously, it may cause a delay. If the industrial Ethernet is used, it also needs to carry out the corresponding communication format conversion chip, so based on the above considerations, the CAN bus is finally selected for the communication between the upper and lower computers, and its circuit is shown in Fig. 10. In addition to communicating with the upper computer, the CAN interface is also used to communicate with the encoder. TMS320F28335 has two CAN interface channels. To make full use of the peripherals of TMS320F28335, CAN-A is selected to communicate with the upper computer and CAN-B is chosen to communicate with the encoder.

**Fig. 11:**
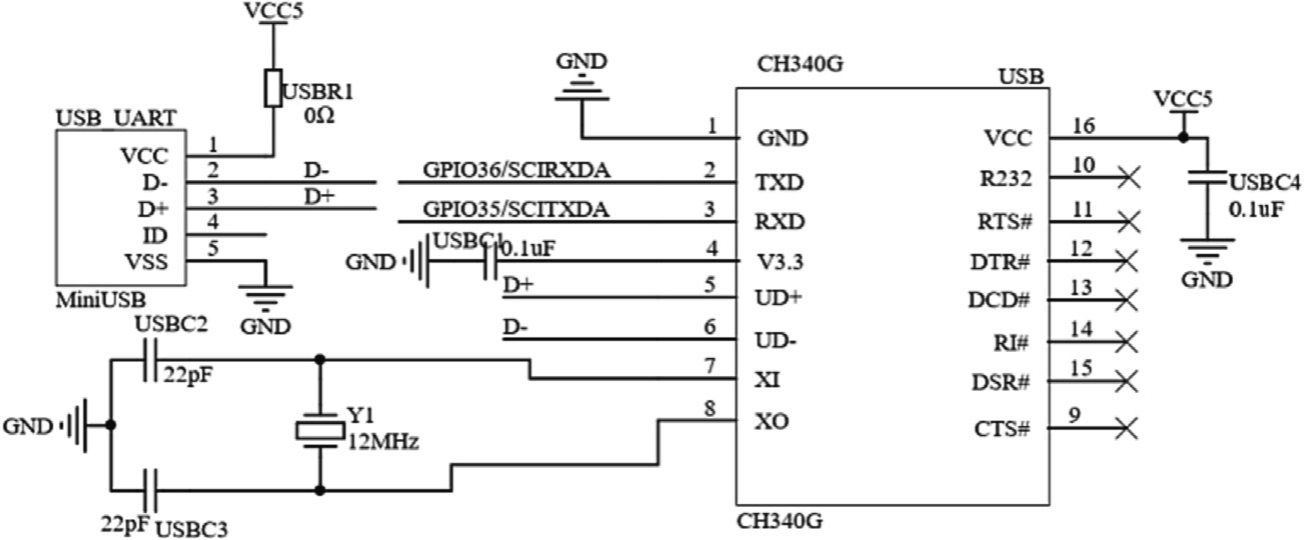
The serial communication module schematic.

At the same time, to realize the external simulation mode in Simulink automatic code generation, a serial communication circuit is designed, as shown in Fig. 11. The controller recognizes the communication between computer USB (Universal Serial Bus) and controller through the CH340 chip, which is convenient for users to monitor sensor data and adjust control parameters in real-time.

**Fig. 12:**
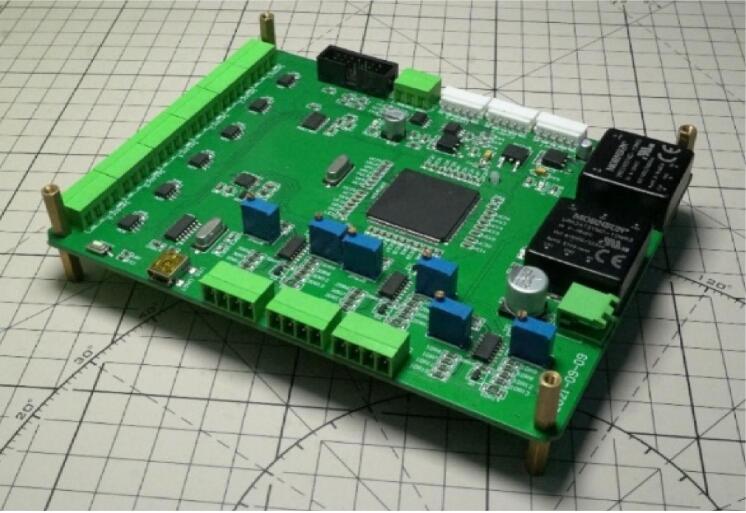
The photo of Control PCB.

**Fig. 13:**
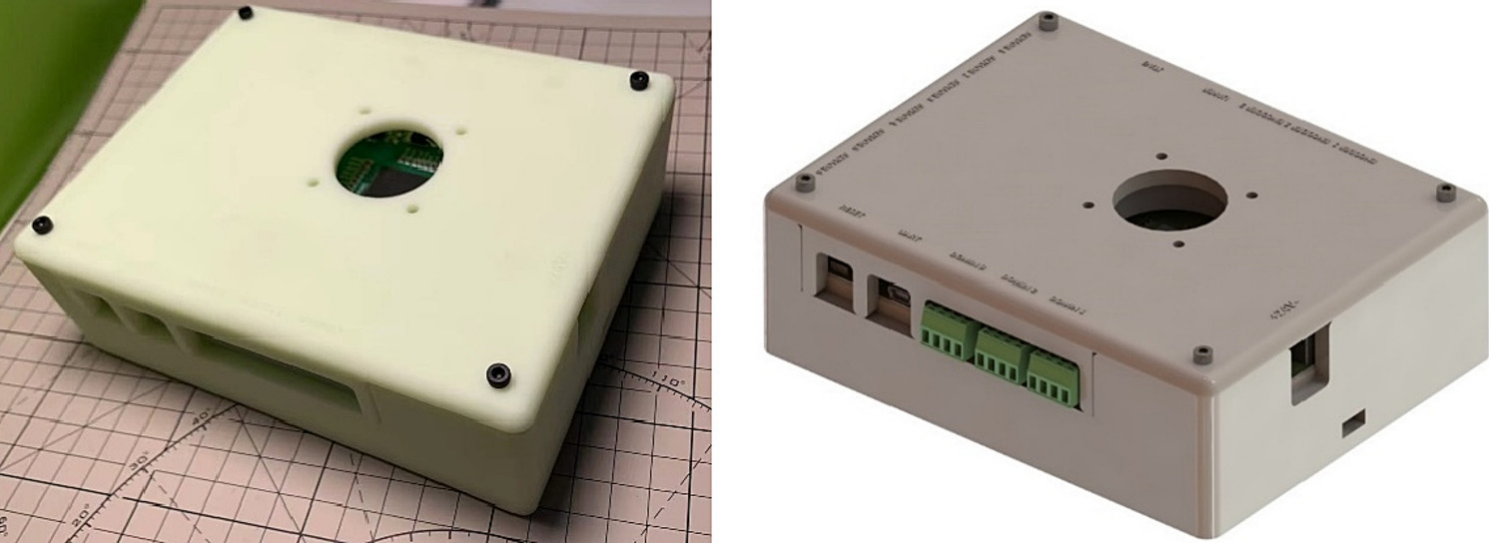
The rendering and photo of Control PCB with the protective shell.

Considering the safety of the installation and the heating of the printed circuit board, after the assembly of the printed circuit board (Fig. 12), it needs to be installed in a protective shell with a cooling fan. The shell can protect the circuit board. At the same time, the cooling fans installed on both sides of the MCU can heat the MCU well and make the MCU run more efficiently. Therefore, the protection shell (Fig. 13) is designed.

## Design files

Design files summary:

• **Quadruped robot controller.PRJPCB:** Altium Designer PCB project file. The PCB project can be viewed and edited if desired.
• **Quadruped robot controller_SCH.SCHDOC:** Altium Designer PCB schematic file. The PCB schematic can be viewed and edited if desired.
• **Quadruped robot controller_SCHLIB.SCHLIB:** Altium Designer PCB schematic library file.
• **Quadruped robot controller_PCB.PcbDoc:** Altium Designer PCB layout file. The PCB layout can be viewed and edited if desired.
• **Quadruped robot controller_PCBLIB.PcbLib:** Altium Designer PCB library file.
• **Quadruped robot controller_PCB.STEP:** 3D model of the PCB assembly.
• **Protective shell (base).STEP:** STEP-File to 3D-print the base of the protective shell. The shell can be viewed and edited if desired.
• **Protective shell (cover).STEP:** STEP-File to 3D-print the cover of the protective shell. The shell can be viewed and edited if desired.

## Bill of materials

Bill of materials summary.

Controller Board:

Link:

https://data.mendeley.com/datasets/zym6bwk37h/2.

Protective shell:

## Build instructions

### Printed circuit board building instructions

**Fig. 14:**
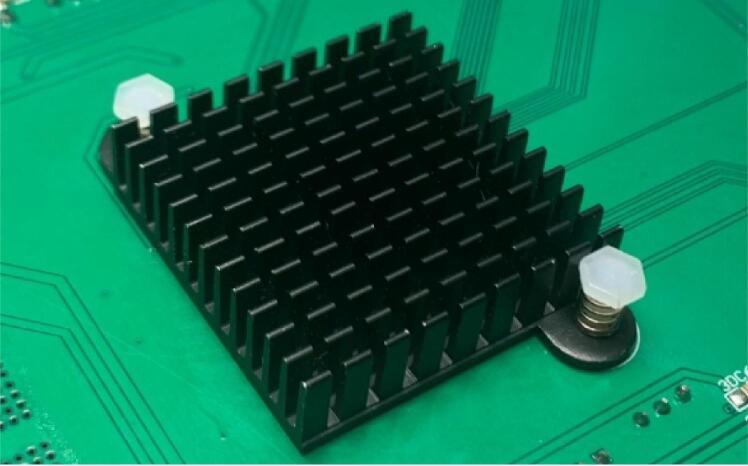
The cooler on the back of MCU.

Use a hot air soldering iron or reflow oven for surface mount device assemblies to populate the printed circuit board. When using this method to weld components, you need to apply solder paste and flux on the bonding pad first, place the components with tweezers, heat the components vertically at the position where the circuit board is soldering components with a hot-air gun, and preferably maintain a height of at least 3 cm to melt the solder paste and flux, and remove the hot-air gun immediately after the components are connected to the circuit board. After all, components are welded, use plate washing water to clean the excess flux on the circuit board. It is recommended to solder operational amplifiers, 0805 footprint capacitors or resistors, 0603 footprint capacitors or resistors, and various types of chips before soldering more significant SMD (Surface Mounted Devices) components and inline type components. Because larger chips or in-line components may make the circuit board uneven, making it difficult to solder other components. Solder through-hole assemblies using soldering iron and solder. Install board spacers or brackets at the four corners of the PCB to avoid any unwanted shorts to potentially conductive surfaces, or use the provided 3D printed protective case for retention and support. The front side of the board has various components of different heights to be soldered, and it is recommended that these components be soldered from shortest to tallest; the back side of the board has only some SMD ceramic capacitors, which can be soldered with limited options. After completing all the welding, you can install the heat sink on the back of the MCU (Fig. 14).

### Protective shell building instructions:

**Fig. 15:**
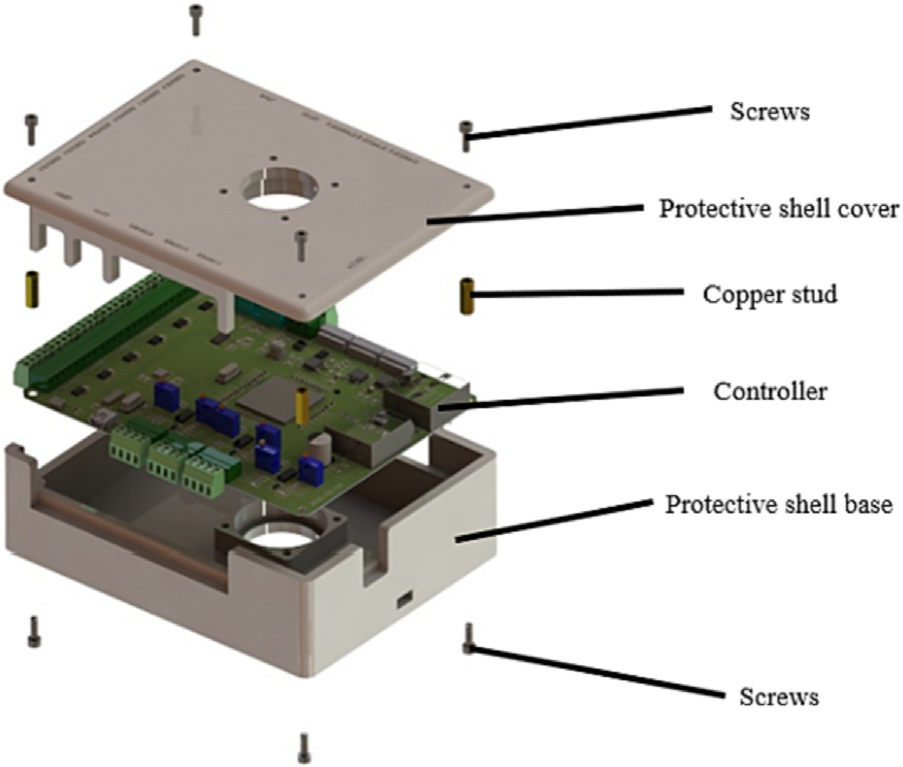
The order of protective installation.

The M3 copper pillar, M3 bolt, 4 cm*4 cm Delta cooling fan, and 3D printed protective shell base and covered included in the BOM table need to be used. The order of installation is shown in Fig. 15. Before the above installation, two 4 cm*4 cm Delta cooling fans need to be installed on the protective shell base and protective shell cover. Then the whole assembly is carried out.

## Operation instructions

**Fig. 16:**
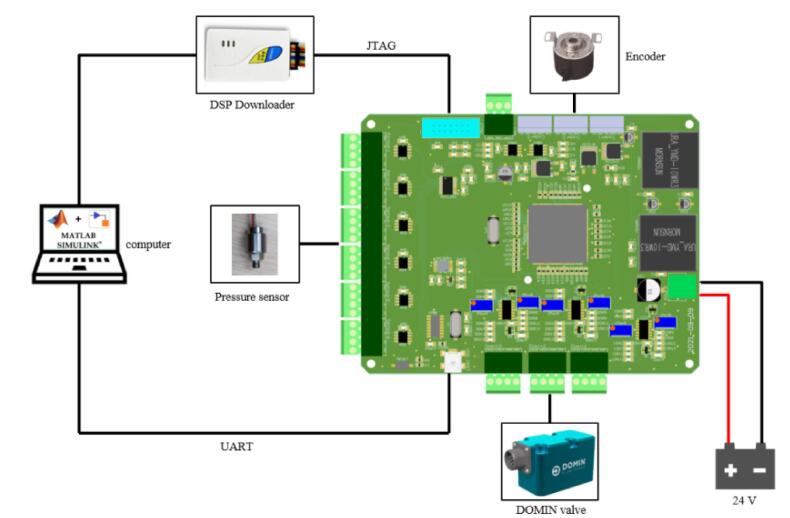
The external interface location.

The operation instruction of printed circuit board:

The controller board can be used generally after connecting the 24 V stable external power supply, and the connection position of other interfaces is shown in Fig. 16. After the 24 V power supply is connected, three LEDs will light up, indicating the regular operation of 15 V, 10 V and 3.3 V power supply respectively. If the three LEDs do not light typically, check the circuit of the power supply part. The four lines of each pressure sensor interface are from left to right: GND, Signal−, Signal+, and 10 V. The five lines of each encoder interface are from left to right: CANL, CANH, NUL, GND, and 24 V. The four lines of each servo valve interface are from left to right: 24 V, Signal+, Signal−, GND. After the servo valve interface is connected, the potentiometer needs to be adjusted to the proper valve so that the DAC chip DAC8554 can output the corresponding 4–20 mA analog current signal. The potentiometers MAXR and MINR correspond to the maximum and minimum values of the output analog current, respectively.

### Programming

As the design of embedded systems becomes increasingly complex, how to quickly extend the developed algorithm to system development has become a significant challenge. To solve this bottleneck, MathWorks deals with the development concept of MBD (Model-Based Design), describes the physical prototype of the system with the Simulink model, and simulates the system in the simulation environment, which can be improved and change over time. The core idea is an executable specification, rapid control prototype design, early verification, and automatic code generation, which can significantly improve the efficiency of system development and gradually become an essential means of embedded development.

**Fig. 17:**
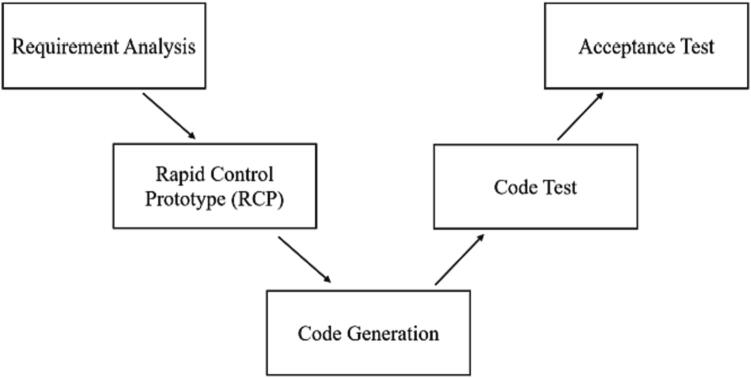
The V-shaped development process.

The whole design and development process adopt the “V-shaped development process.”(Fig. 17) Firstly, analyze the requirements of the entire system, determine the parameters of the controlled object, then design the control strategy to be adopted by the controlled object, and carry out a rapid control prototype design for the controlled object, that is, establish the simulation model, and then carry out the simulation test of the control algorithm to verify the feasibility of the control algorithm; After the algorithm simulation, the code is generated, which is developed on the actual controller hardware, and then the code test and acceptance test are carried out to test the substantial control effect [17], [18].

TI and MathWorks, Inc. cooperate to optimize the development environment for MSP430, C2000, and other series of MCU products so that engineers using MSP430 series and F28x series DSP real-time control microprocessors can adopt a model-based design approach to achieve the entire development chain from requirements analysis to code verification. C2000 series hardware support package contains many standard functional modules, such as GPIO (General-Purpose Input output), CAN, SPI, etc. You can achieve rapid code generation through the corresponding modules, combined with the control algorithm, and then quickly arrange to the target controller.

The software design of this control board is based on this modular set of automatically generated code. To use automatic code generation, install MATLAB r2018a (9.4.0.813654), and then select MATLAB to obtain the hardware support package. Locate the embedded coder support package for Texas Instruments C2000 processors and install the hardware support package. After installation, enter the setting interface to set the hardware package support devices and install the three plug-ins TI Code Composer Studio, TI controlSUITE, and TI C2000Ware, according to the subsequent prompts.

**Fig. 18:**
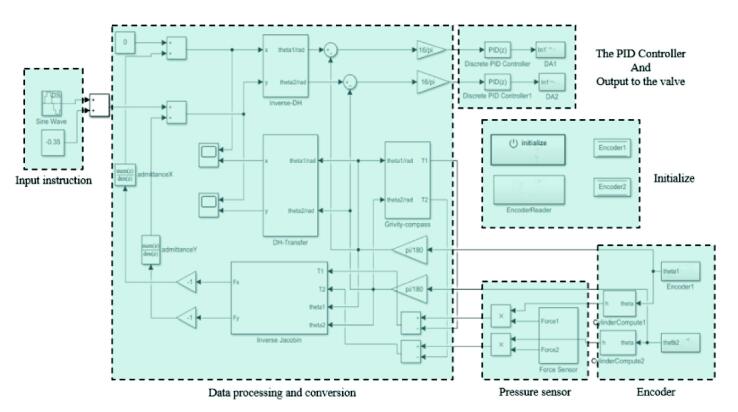
The basic position control framework of this control board.

**Fig. 19:**
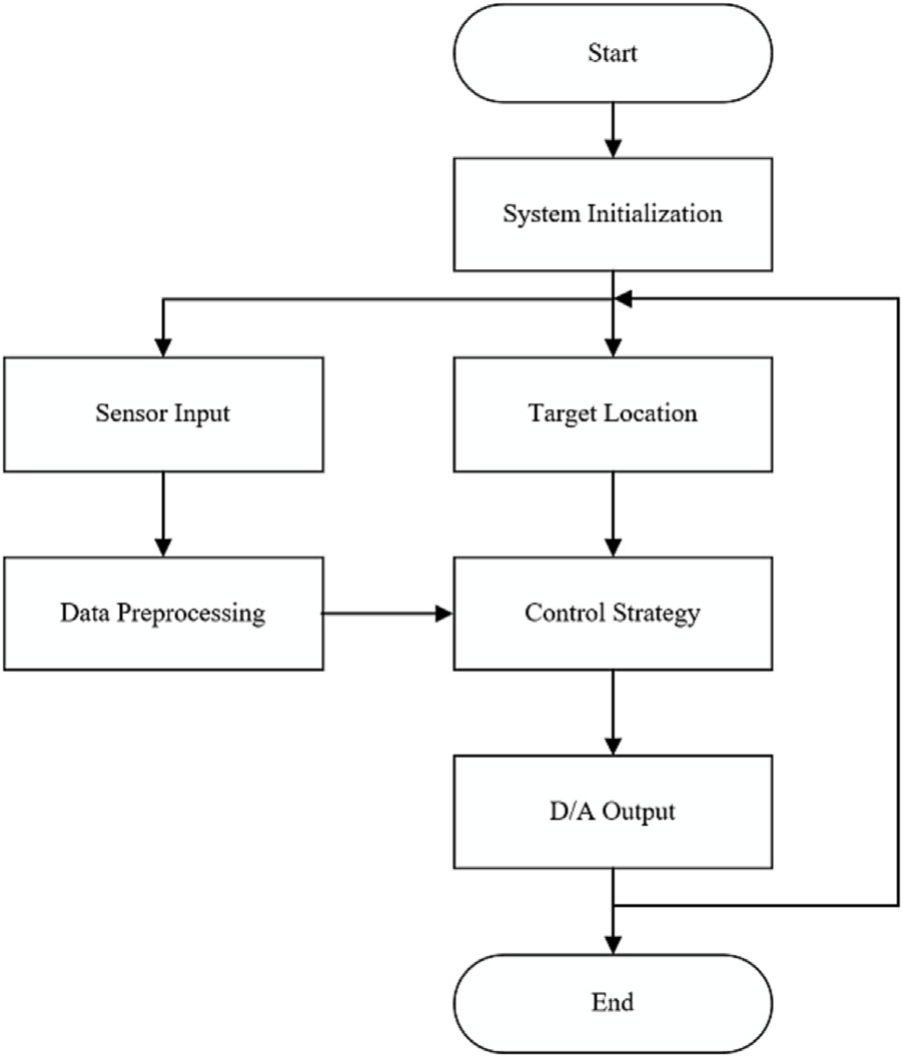
The software execution process of this control board.

After all the above plug-ins and software configurations are completed, you can use the relevant modules in the embedded coder support package for Texas Instruments C2000 processors Library in the new Simulink file for software design. In software design, the external simulation mode can achieve real-time data observation and facilitate data extraction. This method can add a human–machine interface through the dashboard module to provide real-time data for users. Fig. 18 shows the basic software framework of this control board, and Fig. 19 shows the software execution process. This framework mainly contains: the initialize part, the input instruction, the data processing and conversion, the input from the encoders and the pressure sensors, the PID controller, and the output to the servo valves.

**Fig. 20:**
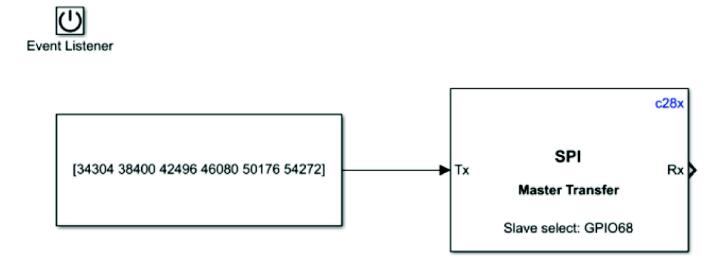
The initialization operation of the MAX1300AUG.

MAX300AUG communicates with the MCU through SPI communication. Because DSP28335 has only one SPI channel, and the datasheet sends 8-bit data and receives 16-bit data. According to the timing sequence diagram in the datasheet, MAX1300AUG will continuously read a channel twice, and the last four bits are 0000 each time; it always reads 0 for the first time, which needs to be discarded. The chip can read the correct data for the second time. Moreover, although the rising edge reception and falling edge transmission shown in the datasheet need to be delayed by half a clock cycle, it will reduce the accuracy of the read data by half. Considering the delay, the number of bits of configuration data is 16 bits. The chip's initialization operation configures the range of each channel, which only needs to be configured once (Fig. 20). However, once the CS chip selection floats, the chip will reset.

**Fig. 21:**
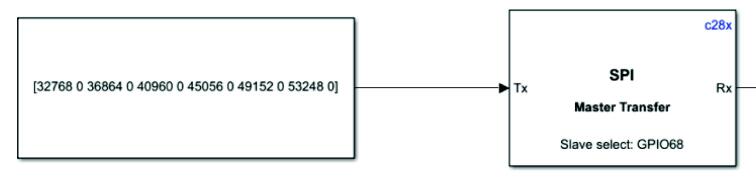
The program example of reading data through MAX1300AUG.

The complete data reading occurs in 32 clock pulses. The data reading and transmission of DSP can be said to be carried out simultaneously, so the reception is also carried out while sending the read instruction. The datasheet shows the first received data is 0; the second time is the actual reception. At this time, it doesn't matter what you send, you can receive the correct data. Therefore, you can see that the second transmission in the instruction is 0. You can send the data continuously if you want to receive it multiple times. Fig. 21 is an example of a group of data reading. 32,768 (1000 0000) is the channel selection, and the following 0 (0000) is the content sent when the correct data is received.

**Fig. 22:**
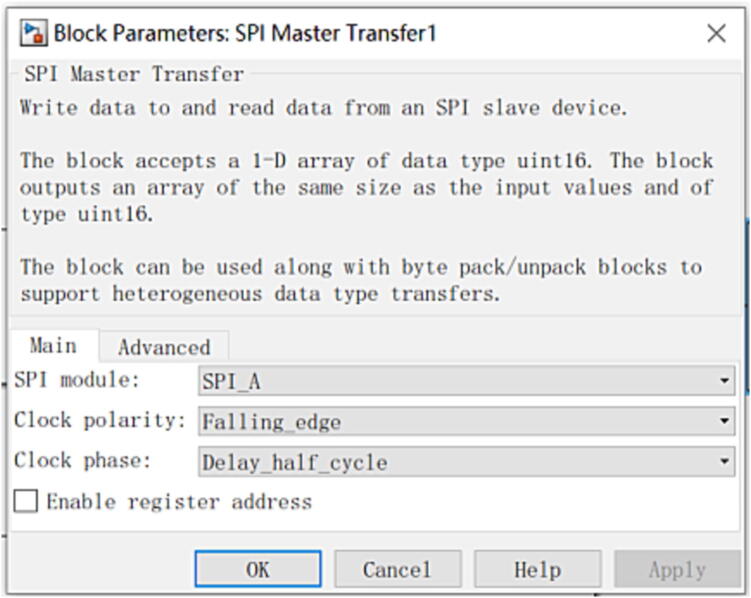
The SPI module setting interface in Simulink.

**Fig. 23:**
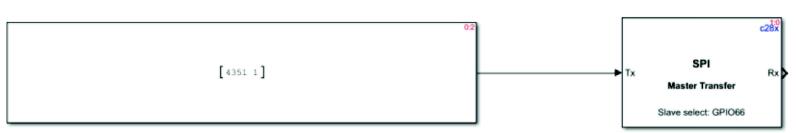
The program example of sending data to DAC8554.

DAC8554 chip also communicates with MCU through SPI. Because the SPI module in the automatic code generation module used must input the data format of uint16 (Fig. 22). Dac8554 needs to read 24bit data, so the first 24 bits input according to the data manual are reserved, and output by the register, and the remaining 8 bits are discarded. Fig. 23 is an example of the program.

## Validation and characterization

**Fig. 24:**
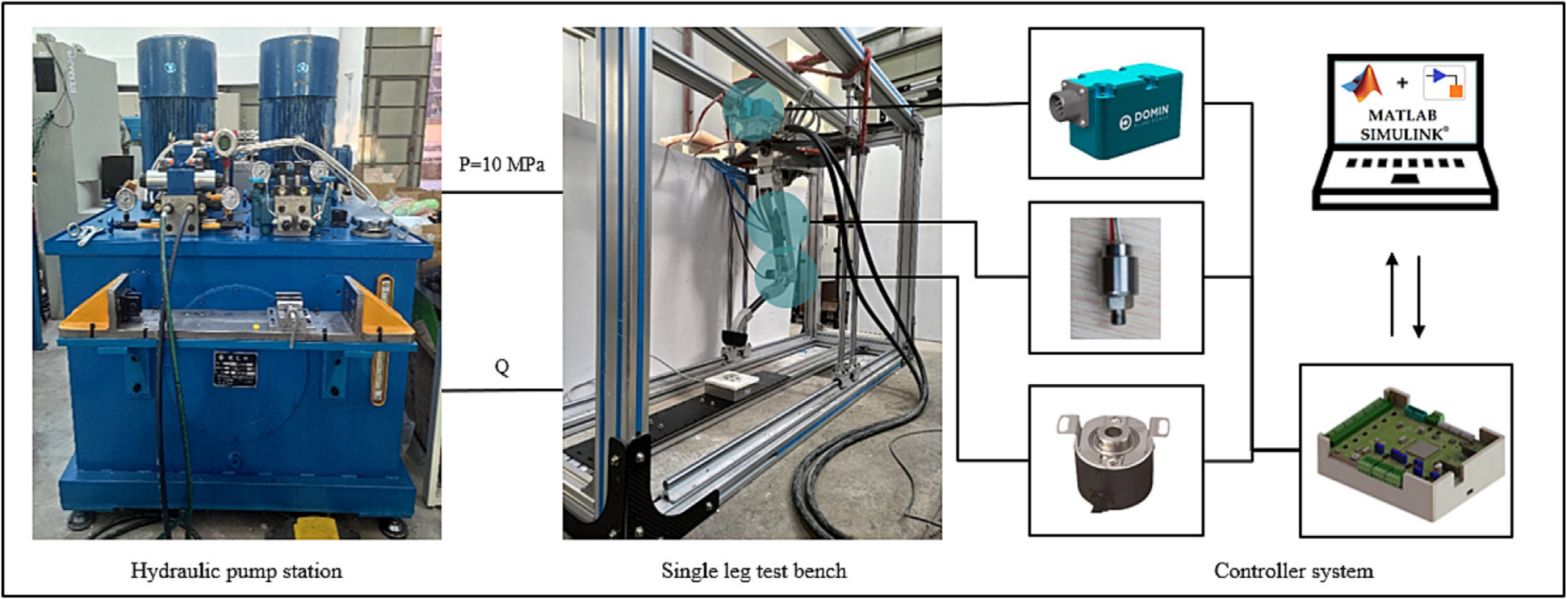
The ‘Spurlos’ hydraulic single leg test bench.

This experiment finally evaluates the control performance of the controller in the single-leg position control according to the lag phase difference of the two joint angles in the track tracking. To verify the function of the controller, take the Spurlos hydraulic single leg test-bed (Fig. 24) as the control object, debug the controller in the EXTERNAL simulation mode of Simulink, and make the foot end move according to the established trajectory. The Spurlos single-leg test bench comprises a hydraulic pump station and a single-leg device. The hydraulic pump station provides a 0–25 MPa pressure power supply for the whole single-leg test bench system, with a maximum flow of 40 L/min. The single-leg test bench is composed of a mechanical structure with two degrees of freedom, hip joint flexion and extension, and knee joint flexion and extension of one leg. The single-leg system allows lateral and longitudinal translation. This experiment finally evaluates the control performance of the controller in the single-leg position control according to the lag phase difference of the two joint angles in the track tracking, stable control requires that the phase difference be kept within 5°.

**Fig. 25:**
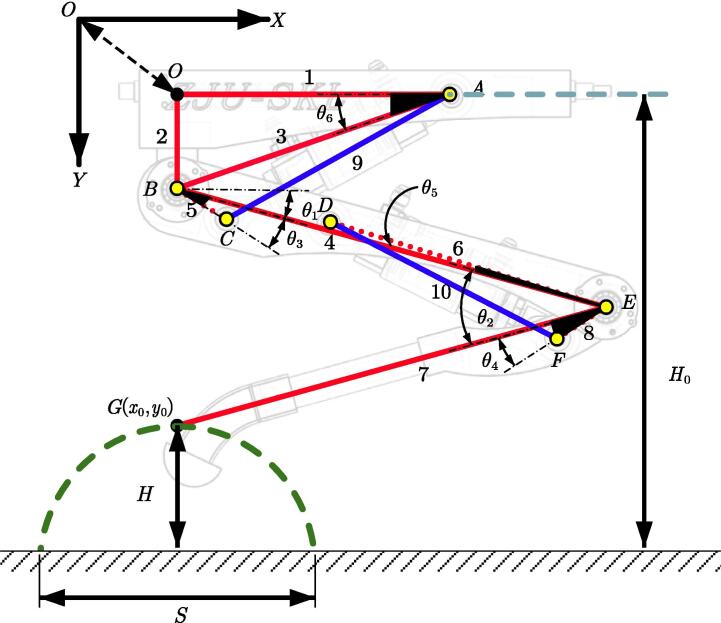
The structure and parameters of Spurlos’ single leg.

Before starting the experiment, the sensor and servo valve need to be zeroed. This experiment is only for foot-end position control. It needs to use communication to read the angle signal of the encoder and SPI communication to control the servo valve. Each joint's position and motion control adopt the PID algorithm to realize the fixed step trajectory motion of the foot endpoint. The structure and specific parameters of the single leg test bench are shown in Table 4 and Fig. 25, and the set trajectory parameters of the foot end are shown in Table 5. The corresponding blocking motion trajectory function is established by using the quintic polynomial trajectory:

**Table 4:** The parameters of Spurlos’ single leg.

	l1	l2	l3	l4	l5	l6	l7	l8	l9, *l*10
Length(mm)	216.00	72.30	228.00	350.00	45.00	228.00	350.00	45.00	Variable

	$\theta_{1}$	$\theta_{2}$	$\theta_{3}$	$\theta_{4}$	$\theta_{5}$	$\theta_{6}$
Angle(°)	15.00–100.00	30.00–115.00	15.00	17.00	1.50	18.50

**Table 5:** The foot trajectory parameters.

Parameters	Step *S* (m)	Period *T* (s)	Step height *H* (m)	Height *H0* (m)	Duty ratio *β*
Value	0.30	1	0.10	0.55	0.5

The trajectory of the quintic polynomial is:

(3) $$P\left(t\right)=\left(6t^{5}-15t^{4}+10t^{3}\right)\left(P_{f}-P_{0}\right)+P_{0}$$

In which: $P_{0}$ is initial position coordinates, $P_{f}$ is moving target point coordinates.

The foot end trajectory is:

(4) $$x\left(t\right)=\left\{\begin{array}{c} 0.3(6t^{5}-15t^{4}+10t^{3})-0.5S,0<t⩽0.5\\ 0.3(6{\left(1-t\right)}^{5}-15{\left(1-t\right)}^{4}+10{\left(1-t\right)}^{3})-0.5S,0<t⩽1 \end{array}\right)$$

(5) $$y\left(t\right)=\left\{\begin{array}{c} 0.55-0.1(6t^{5}-15t^{4}+10t^{3}),0<t⩽0.25\\ 0.55-0.1(6{\left(0.5-t\right)}^{5}-15{\left(0.5-t\right)}^{4}+10{\left(0.5-t\right)}^{3}),0.25⩽t⩽0.5\\ 0.55-0.1(6{\left(t-0.5\right)}^{5}-15{\left(t-0.5\right)}^{4}+10{\left(t-0.5\right)}^{3}),0.5⩽t⩽0.75\\ 0.55-0.1(6{\left(1-t\right)}^{5}-15{\left(1-t\right)}^{4}+10{\left(1-t\right)}^{3}),0.75⩽t⩽1 \end{array}\right)$$

**Fig. 26:**
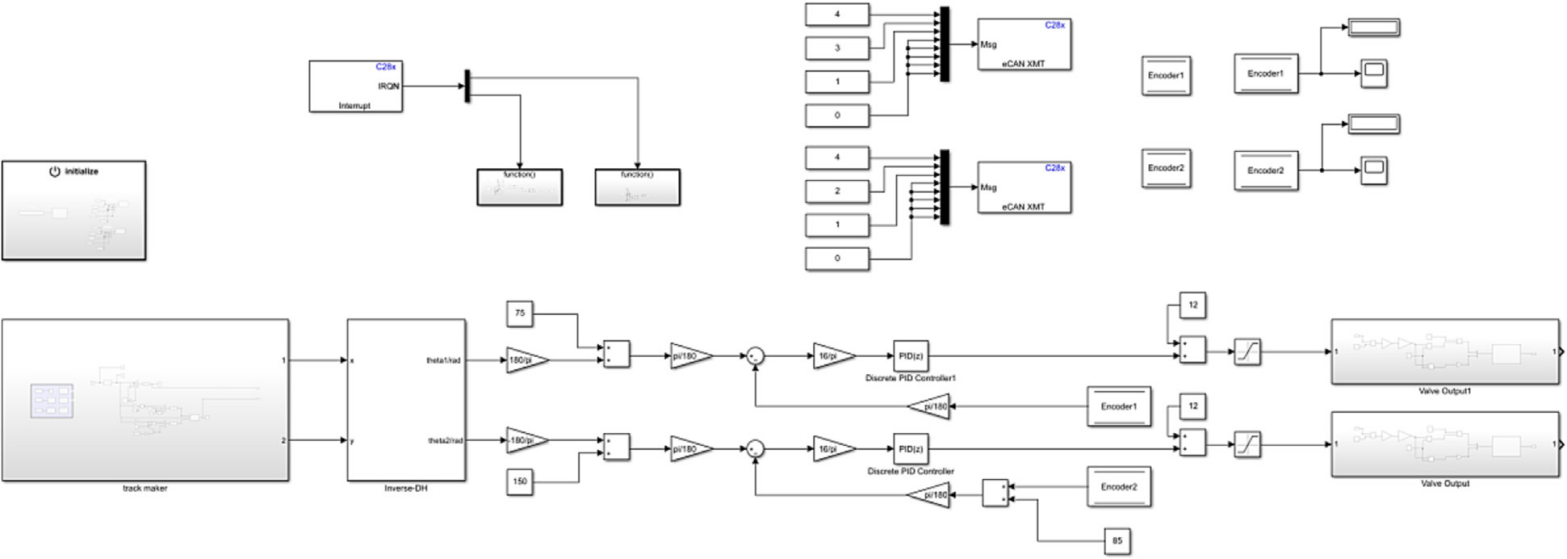
The experimental Simulink software block diagram.

**Fig. 27:**
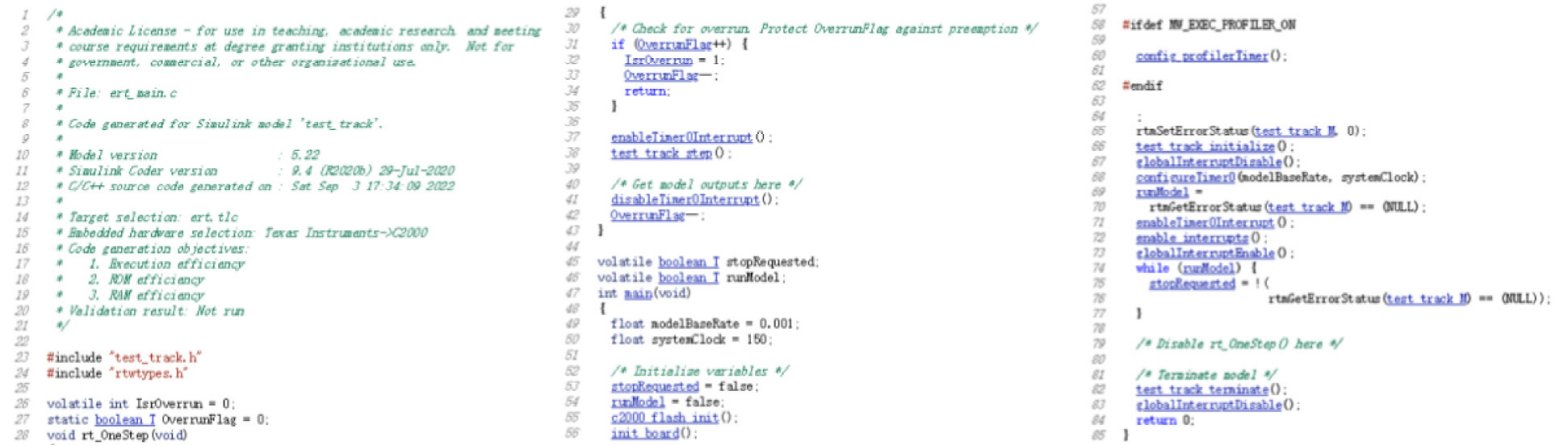
The experimental code ‘main.c’.

The block diagram of the experimental Simulink software is shown in Fig. 26. Use the automatic code generation method described in the previous section to generate an executable file (Fig. 27), which is downloaded to the controller through the DSP downloader.

**Fig. 28:**
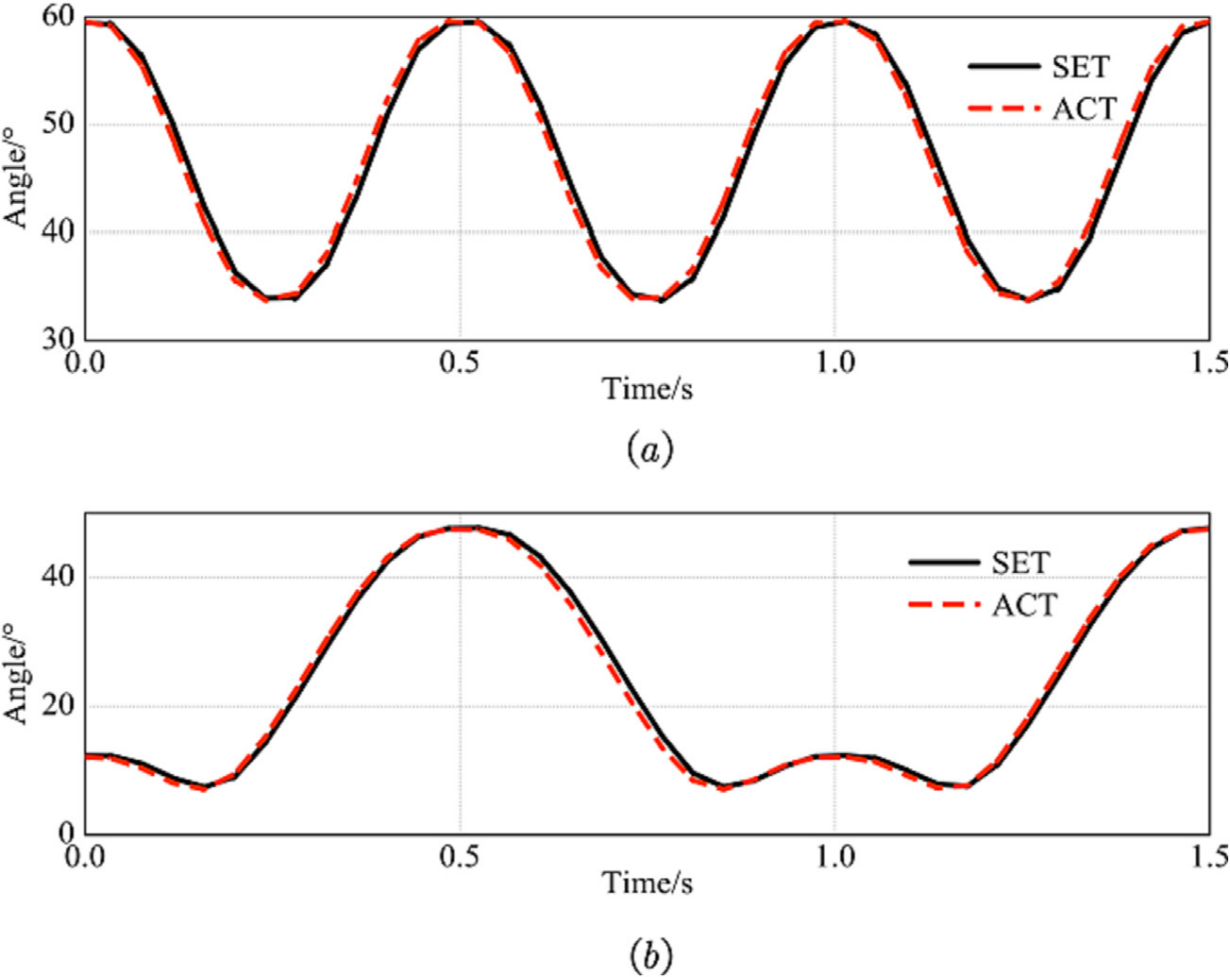
The result of the test. a. Knee joint angle. b. Hip joint angle. The solid black line is the set value, and the red dotted line is the actual value. (For interpretation of the references to colour in this figure legend, the reader is referred to the web version of this article.)

After downloading, open the hydraulic pump station, control the system pressure to 10 MPa, and conduct the motion test. To make the quadruped robot better adapt to different terrain, the robot leg needs a certain degree of flexibility to protect the portion [19], [17], [20], [21], [22]. This is also one of the directions of future research. This paper uses the admittance control strategy based on the position inner loop for single branch control [23]. Fig. 28 shows the difference between the hip joint angle in the test and the set value and actual value of the knee joint angle. The phase difference of joint angle tracking is 1.26°, within 5°. It is proved that the position of the foot tip can move stably under the established trajectory. This program is also included in the open-source library file.

The future practice direction is mainly to implement and test other control algorithms except for the PID algorithm of the controller in the single-leg module. The gap between this study and other studies is that the torque loop control experiment of the single leg experimental platform has not been tested, which is also one of the future directions that this study will study.

## Conclusion

This paper proposes a design scheme for the bottom controller for the distributed control hydraulic quadruped robot. The circuit design, building guide, program design, and experimental verification of the controller are described in detail. In the experimental validation, researchers can use this controller to control the position of the single leg platform of the Spurlos hydraulic quadruped robot, and the expected control effect is achieved.

Compared with previous studies, the controller designed in this study is the bottom controller of the distributed control hydraulic quadruped robot, which is suitable for controlling the single leg of the hydraulic quadruped robot with three degrees of freedom (hip abduction/adduction, hip flexion/extension, and knee flexion/extension). The program design of this controller uses the MBD development mode with higher development efficiency, which is more convenient for developers to develop programs and verify algorithms on the hydraulic quadruped robot.

The design and implementation of this controller in this study will provide a relatively mature bottom controller scheme for the split control of hydraulic quadruped robots, facilitate the development of control programs and the verification of control algorithms by other researchers of hydraulic quadruped robots, and promote the research and development of the field of hydraulic quadruped robots.

This study's most significant limitation lies in the control signals of sensors and servo valves. If this controller is to be used for the bottom control of the hydraulic quadruped robot, it needs to meet the consistency of the control signals of sensors and servo valves. At the same time, it is also necessary to design more efficient and fast communication methods to improve the controller's data transmission and data processing capacity. Therefore, this research's future direction is to make the controller have higher versatility and integration.

## CRediT authorship contribution statement

**Lizhou Fang:** Writing – original draft, Data curation, Visualization. **Junhui Zhang:** Resources, Funding acquisition, Writing – review & editing. **Huaizhi Zong:** Software, Validation. **Ximeng Wang:** Supervision, Project administration. **Kun Zhang:** Conceptualization, Methodology. **Jun Shen:** Formal analysis, Investigation. **Zhenyu Lu:** Writing – review & editing, Supervision.

## Declaration of Competing Interest

The authors declare that they have no known competing financial interests or personal relationships that could have appeared to influence the work reported in this paper.
